# Prediction of half-marathon race time in recreational female and male runners

**DOI:** 10.1186/2193-1801-3-248

**Published:** 2014-05-16

**Authors:** Beat Knechtle, Ursula Barandun, Patrizia Knechtle, Matthias A Zingg, Thomas Rosemann, Christoph A Rüst

**Affiliations:** Institute of General Practice and for Health Services Research, University of Zurich, Zurich, Switzerland; Gesundheitszentrum St. Gallen, Vadianstrasse 26, 9001 St. Gallen, Switzerland

**Keywords:** Running, Performance, Body fat, Training

## Abstract

Half-marathon running is of high popularity. Recent studies tried to find predictor variables for half-marathon race time for recreational female and male runners and to present equations to predict race time. The actual equations included running speed during training for both women and men as training variable but midaxillary skinfold for women and body mass index for men as anthropometric variable. An actual study found that percent body fat and running speed during training sessions were the best predictor variables for half-marathon race times in both women and men. The aim of the present study was to improve the existing equations to predict half-marathon race time in a larger sample of male and female half-marathoners by using percent body fat and running speed during training sessions as predictor variables. In a sample of 147 men and 83 women, multiple linear regression analysis including percent body fat and running speed during training units as independent variables and race time as dependent variable were performed and an equation was evolved to predict half-marathon race time. For men, half-marathon race time might be predicted by the equation (r^2^ = 0.42, adjusted r^2^ = 0.41, SE = 13.3) half-marathon race time (min) = 142.7 + 1.158 × percent body fat (%) – 5.223 × running speed during training (km/h). The predicted race time correlated highly significantly (r = 0.71, p < 0.0001) to the achieved race time. For women, half-marathon race time might be predicted by the equation (r^2^ = 0.68, adjusted r^2^ = 0.68, SE = 9.8) race time (min) = 168.7 + 1.077 × percent body fat (%) – 7.556 × running speed during training (km/h). The predicted race time correlated highly significantly (r = 0.89, p < 0.0001) to the achieved race time. The coefficients of determination of the models were slightly higher than for the existing equations. Future studies might include physiological variables to increase the coefficients of determination of the models.

## Background

Marathon running is still of high popularity, however, half-marathon running is of higher popularity than marathon running. Running USA presents an annual report regarding the development in marathon and half-marathon running in the United States of America. In the actual marathon report 2013, the question arose whether the bloom has gone from marathon running (Running USA’s annual marathon report). This question was asked after a modest growth in US-American marathon finishers in 2011 compared to previous years. For the first time since 2001, the estimated number of US-American marathon finishers declined from a record of 518,000 finishers in 2011 to 487,000 finishers in 2012 with a 6% decrease. However, like in 2001, most of the decline was attributed to a unique situation. In 2001, 9/11 lead to a fall in marathon participation, and in 2012, the cancellation of the ‘New York City Marathon’, the world's largest marathon with more than 47,000 finishers or 9% of the 2011 overall finishers.

In contrast, since 2003, the half-marathon distance was the fastest growing race distance in road running in the United States of America, and for seven consecutive years from 2006 to 2012, the number of half-marathon finishers has grown by 10% or more each year (Running USA’s annual half-marathon report). No other road distance came close to this growth rate during the second running boom in 1994. Since 2000, the number of US-American half-marathon finishers has nearly quadrupled from 482,000 to 1,850,000 finishers with an impressive 284% increase. Regarding the sexes, for the first time in history, 60% of US-American half-marathon finishers were women with ~1,110,000 finishers. Although the percentage of male US-American half-marathon finishers has declined to 40%, there was a record of 740,000 male finishers in US-American half-marathons in 2012.

Both half-marathon and marathon runners need their specific preparation (Zillmann et al. 2013). However, half-marathoners showed differences in both anthropometry and training characteristics compared to marathoners that could be related to their lower training volume, most probably due to the shorter race distance they intended to compete in. Both groups of athletes seemed, however, to profit from a low body fat and a high running speed during training for fast race times (Zillmann et al. 2013).

Recent studies tried to find predictor variables for half-marathon race time for recreational female (Knechtle et al. 2011a) and recreational male (Rüst et al. 2011) runners. A study including 42 female half-marathoners found that half-marathon race time might be partially predicted by the equation race time (min) = 166.7 + 1.7 × (midaxillary skinfold, mm) - 6.4 × (running speed during training, km/h) (Knechtle et al. 2011a). A study investigating 84 recreational male runners found that half-marathon race time might be partially predicted by using the equation race time (min) = 72.91 + 3.045 × (body mass index, kg/m^2^) - 3.884 × (running speed during training, km/h) (Rüst et al. 2011). For both women and men, the equation included an anthropometric and a training variable where running speed during training seemed to be an important predictor variable for both sexes. However, regarding anthropometric characteristics in half-marathoners, an actual study showed that for both women and men, percent body fat and running speed during training sessions were related to half-marathon race times when corrected with co-variables after multi-variate regression analyses (Friedrich et al. 2014).

This actual finding raises the question whether the equations for female (Knechtle et al. 2011a) and male (Rüst et al. 2011) recreational half-marathoners should be reconsidered since for women, midaxillary skinfold was the anthropometric predictor variable (Knechtle et al. 2011a) and for men, body mass index was predictive (Rüst et al. 2011). The aim of the present study was to revise and improve the existing equations to predict half-marathon race time in a larger sample of runners by using percent body fat and running speed during training sessions as the most important predictor variables (Friedrich et al. 2014).

## Methods

### Ethics

The study was approved by the Institutional Review Board for use of Human Subjects of the Canton of St. Gallen, Switzerland. All athletes participating in the study were informed of the experimental procedures and gave their informed written consent.

### Subjects

In the ‘Basel Marathon’ held in Basel, Switzerland, athletes can run either a half-marathon or a full marathon. In the half-marathon, the competitors have to run one lap of 21.0975 km on asphalt with a total altitude of 200 m. All female and male half-marathoners participating in the 2010 and 2011 edition were informed via electronic newsletters sent by the organizer three months before the race. Information about the planned investigation was also provided on the race website. Participating athletes were included only once and recruited continuously during two consecutive years from 2010 to 2011 in order to increase the sample size. Course and nutrition for athletes and general weather conditions were identical in both years.

### Measurements and calculations

The participants were asked to record the distance and time of each training unit during the three months prior to the race. The investigator provided an electronic file where the subjects could insert each training unit with distance in kilometres (km), duration in minutes (min) and running speed in kilometres per hour (km/h). The investigator calculated the mean weekly training hours, the mean weekly training kilometres and the mean running speed during training in the pre-race preparation. The participants were also asked to provide the number of completed half-marathons and their personal best time in half-marathon where the personal best time in half-marathon was defined as the fastest time ever achieved during life in half-marathon running.

On the afternoon of the day before the race, anthropometric characteristics such as body mass, body height, and the thicknesses of eight skinfolds (*i.e.* chest, midaxillary, triceps, subscapular, abdomen, suprailiac, thigh, and calf) were measured on the right side of the body. Additionally, circumference of hip was measured. Body mass index was calculated using body mass and body height. Percent body fat was estimated using different anthropometric equations for both women and men. Body mass was measured using a commercial scale (Beurer BF 15, Beurer, Ulm, Germany) to the nearest 0.1 kg and body height was determined using a stadiometer (Tanita HR 001 Portable Height Measure, Tanita Europe, Amsterdam, Netherlands) to the nearest 1.0 cm. All skinfold data were obtained using a skinfold caliper (GPM-Hautfaltenmessgerät, Siber & Hegner, Zurich, Switzerland) and recorded to the nearest 0.2 mm. The skinfold caliper measures with a pressure of 0.1 MegaPascal (Mpa) ± 5% over the whole measuring range. The skinfold measurements were taken once for all eight skinfold sites, and then the procedures were repeated twice more by the same investigator. The mean of the three measurements was used for further calculations. The timing of the skinfold measurement was standardized to ensure reliability. It has been suggested that the best readings are those performed 4 sec after applying the caliper (Becque et al. 1986). The circumference of the hip was measured using a non-elastic measuring (cm) tape (KaWe CE, Kirchner und Welhelm, Germany) to the nearest 0.1 cm.

One trained investigator took all skinfold measurements as inter-tester variability is a major source of error in skinfold measurement. Intra- and inter-rater agreement was assessed from 27 men runners prior to an ultra-marathon, based on measurements taken by two experienced primary care physicians (Knechtle et al. 2010b). Intra-class correlation (ICC) within the two raters was excellent for all anatomical measurement sites and for summary measurements of skinfold thicknesses (ICC > 0.9). Agreement tended to be higher within than between raters, but still reached excellent reliability with ICC = 0.99 (0.99-1.00 95% confidence interval) for the summary measurements of skinfold thicknesses between raters. ICC for investigator 1 versus investigator 1 and for investigator 2 versus investigator 2 for a single skinfold thickness was between 0.98 and 0.99, respectively. ICC was 0.99-1.00 for the sum of seven and eight skinfolds, respectively. For the sum of eight skinfolds for investigator 1, bias (*i.e.* average difference between investigator 1 and investigator 2) was – 0.515 mm, standard deviation of the average difference was 1.492 mm, and 95% limits of agreement were between -3.439 mm and 2.409 mm.

Percent body fat was estimated for women using the formula percent body fat (%) = – 6.40665 + 0.41946 (Σ3SF) – 0.00126 (Σ3SF) ^2^ + 0.12515 (hip) + 0.06473 (age) following Ball et al. (2004a). Σ3SF was taken as the sum of three skinfold (SF) thicknesses (*i.e.* triceps, suprailiac and thigh) and hip was the circumference of the hip. For men, percent body fat was estimated using the anthropometric formula according to Ball et al. (2004b) with percent body fat (%) = 0.465 + 0.180 × (Σ7SF) – 0.0002406 × (Σ7SF)^2^ + 0.0661 × (age), where Σ7SF is the sum of seven skinfold thickness (*i.e.* chest, midaxillary, triceps, subscapular, abdomen, suprailiac, and thigh) in mm and age is in years.

### Statistical analysis

Data were analysed using Analyse-it Software Ltd. (The Tannery, 91 Kirkstall Road, Leeds, LS3 1HS, United Kingdom). Prior to analysis, all data were checked for distribution of normality and are presented as mean ± standard deviation (SD). The coefficient of variation (CV) of performance (CV% = 100 × SD/mean) was calculated for half-marathon race times. Data for women and men were compared using the Mann-Whitney *U*-test. Since Friedrich et al. (2014) reported that both percent body fat and running speed during training units were the best predictors for half-marathon race time in recreational runners, we used a multiple linear regression analysis including percent body fat and running speed during training units as the independent variables and half-marathon race time as the dependent variable and an equation was created to predict half-marathon race time with these two variables. Bland-Altman analysis was used to determine absolute limits of agreement between predicted and effective race times. Correlation analyses were used to investigate the association between effective and predicted race times using the new equations. To investigate a potential correlation between other variables (*i.e.* age, number of completed half-marathons, and personal best time in half-marathon), bi-variate correlation analyses were performed. Statistical significance was set at *p* < 0.05.

## Results

### Performance

A total of 147 men and 83 women completed the races. Men finished the half-marathon within 106.8 ± 17.3 min (CV 16.2%) while running at a mean speed of 12.2 ± 1.9 km/h. They finished within 157 ± 21% of the course record of 1 h 8 min (2011). Women finished within 125.5 ± 17.3 min (CV 13.8%) running at a mean speed of 10.2 ± 1.3 km/h. Expressed in percent of the course record of 1 h 31 min (2010), women finished within 138 ± 18% of the course record. During the race, men were running significantly faster than women (*p* < 0.0001). However, when the performance expressed in percent of the course record was compared between men and women, men were significantly slower than women (*p* < 0.0001).

### Differences between men and women in anthropometric and training characteristics

Men had a higher body mass, a higher body height, and a higher body mass index compared to women (Table 1). Men had a lower skinfold thickness at triceps, thigh and calf site compared to women. At subscapular site, men had a thicker skinfold than women. Men had a lower sum of total skinfold thickness and a lower percent of body fat compared to women. During training, men were running faster, had completed more previous half-marathons and had a faster personal best half-marathon race time compared to women.

**Table 1:** **Comparison of anthropometric and training characteristics between men and women**

	Men (***n*** = 147)	Women (***n*** = 83)
Age (years)	40.2 ± 10.1	38.3 ± 9.2
Body mass (kg)	75.8 ± 8.6	60.1 ± 7.8***
Body height (m)	1.79 ± 0.06	1.66 ± 0.06***
Body mass index (kg/m^2^)	23.3 ± 2.2	21.7 ± 2.3***
Skinfold chest (mm)	9.4 ± 4.2	8.5 ± 4.5
Skinfold midaxillary (mm)	10.6 ± 4.3	10.4 ± 4.5
Skinfold triceps (mm)	8.6 ± 2.8	13.5 ± 4.3***
Skinfold subscapular (mm)	11.3 ± 4.3	10.5 ± 4.5*
Skinfold abdomen (mm)	18.8 ± 9.1	16.9 ± 6.5
Skinfold suprailiac (mm)	20.8 ± 9.4	20.7 ± 8.3
Skinfold thigh (mm)	13.7 ± 6.1	26.4 ± 9.4***
Skinfold calf (mm)	6.7 ± 2.7	10.3 ± 4.8***
Sum of 8 skin-folds (mm)	99.9 ± 35.6	117.3 ± 38.3***
Percent body fat (%)	17.5 ± 4.6	28.4 ± 5.3***
Years as active runner (y)	7.9 ± 8.0	6.1 ± 5.0
Weekly running kilometres (km)	33.7 ± 20.5	33.5 ± 17.0
Minimal weekly running distance (km)	16.2 ± 13.5	15.5 ± 10.1
Maximal weekly running distance (km)	45.2 ± 29.1	41.6 ± 18.5
Weekly running hours (h)	3.9 ± 2.0	3.6 ± 1.8
Number of running training units (n)	3.1 ± 1.3	3.0 ± 1.0
Distance per running training unit (km)	11.3 ± 3.2	10.4 ± 2.9
Duration per running training unit (min)	63.0 ± 16.5	63.5 ± 16.0
Speed during running training units (km/h)	10.8 ± 1.5	9.8 ± 1.5**
Number of completed half-marathons (n)	6 ± 7 (*n* = 122)	5 ± 2 (*n* = 43)*
Personal best time (min)	102 ± 17	115 ± 21**

Results are presented as mean ± SD. * = p < 0.05, ** = p < 0.01, *** = p < 0.001.

### Equations after multi-linear regression analyses

**Figure 1:**
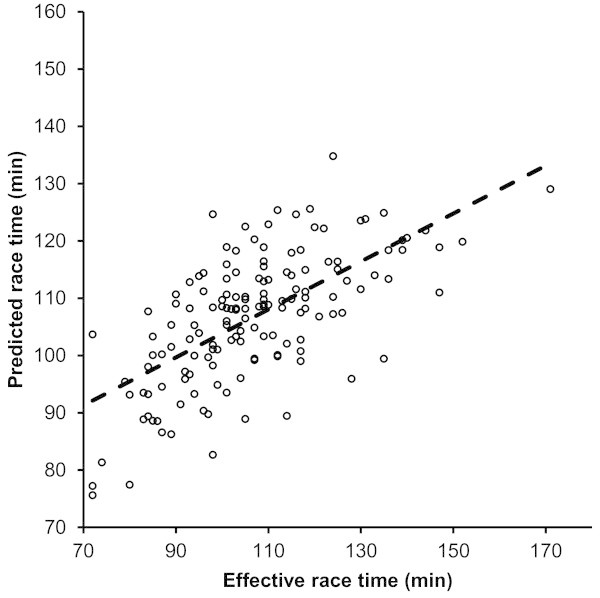
**The predicted half-marathon race time correlated significantly to the achieved half-marathon race time in men.**

**Figure 2:**
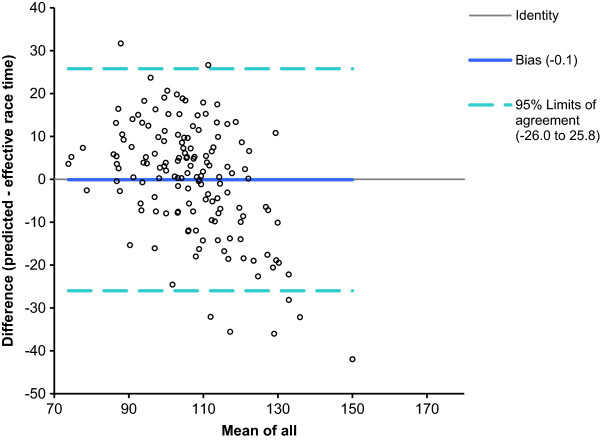
**Bland-Altman plots comparing predicted with effective race time for men.**

For men, half-marathon race time might be partially predicted by the equation (r^2^ = 0.42, adjusted r^2^ = 0.41, SE = 13.3) race time (min) = 142.7 + 1.158 × percent body fat (%) – 5.223 × running speed during training (km/h). Percent body fat explained 24% of the variance (r^2^ = 0.24) and running speed 34% (r^2^ = 0.34). The predicted race time was 106.7 ± 11.2 min and correlated highly significantly (r = 0.71, p < 0.0001) to the achieved race time (Figure 1). Figure 2 shows the level of agreement using Bland-Altman method (95% limits of agreement -26.0 to 25.8 min) between effective and predicted race times.

**Figure 3:**
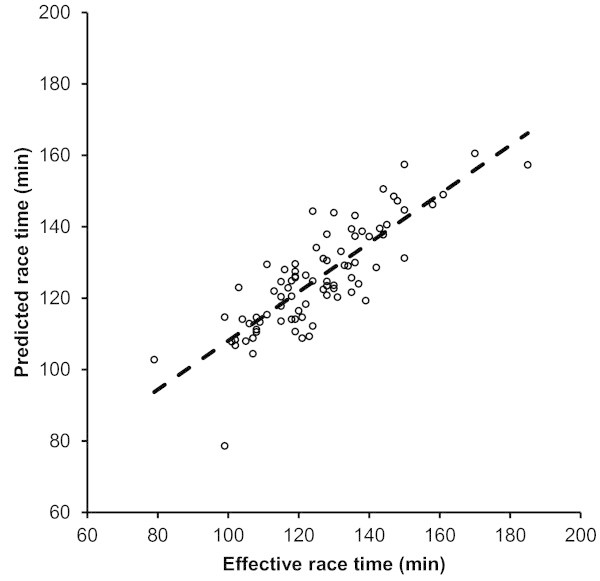
**The predicted half-marathon race time correlated significantly to the achieved half-marathon race time in women**

**Figure 4:**
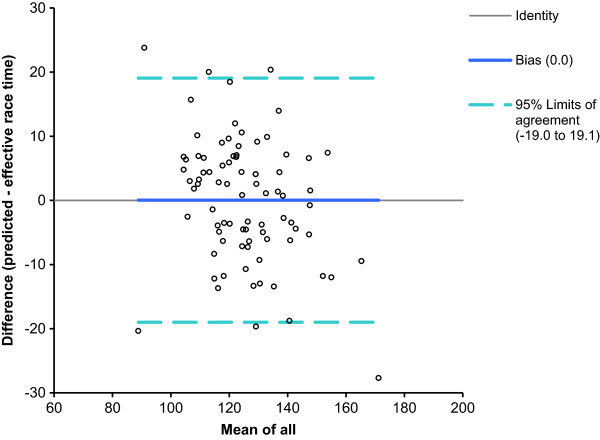
**Bland-Altman plots comparing predicted with effective race time for women**

For women, half-marathon race time might be partially predicted by the equation (r^2^ = 0.68, adjusted r^2^ = 0.68, SE = 9.8) race time (min) = 168.7 + 1.077 × percent body fat (%) – 7.556 × running speed during training (km/h). Percent body fat explained 33% (r^2^ = 0.33) and running speed 59% (r^2^ = 0.59) of the variance. The predicted race time was 125.5 ± 14.3 min and correlated highly significantly (r = 0.89, p < 0.0001) to the achieved race time (Figure 3). Figure 4 shows the level of agreement using Bland-Altman method (95% limits of agreement -19.0 to 19.1 min) between effective and predicted race time.

### Correlation of other variables with race time

In both men and women, age (r = 0.27, p = 0.0010 and r = 0.27, p = 0.015, respectively) and the personal best time in half-marathon (r = 0.85, p < 0.0001 and r = 0.35, p = 0.011, respectively) correlated to race time, but not the number of completed half-marathons (r = -0.13, p = 0.15 and r = -0.08, p = 0.60, respectively).

## Discussion

This study intended to improve the actual equations to predict half-marathon race time for recreational female (Knechtle et al. 2011a) and male (Rüst et al. 2011) half-marathoners in a larger sample by using percent body fat and running speed during training sessions as the most important predictor variables for half-marathon race time (Friedrich et al. 2014).

An interesting finding was that the coefficient of determination of the models was higher in women (r^2^ = 0.68) than in men (r^2^ = 0.42). Interestingly, also in the existing equations for male (Rüst et al. 2011) and female half-marathoners (Knechtle et al. 2011a), the coefficients of determination were similar for women (r^2^ = 0.71) and men (r^2^ = 0.44) although the sample size in the present study was considerably higher. The differences in the coefficients of determination in the models might be explained by differences in anthropometric and training characteristics between women and men. Firstly, men and women showed differences in their anthropometric characteristics. The most important differences were body mass, body height, body mass index, percent body fat, and the sum of eight skinfolds (*e.g.* chest, midaxillary, triceps, subscapular, abdomen, suprailiac, thigh and calf). These findings confirm existing findings. Knechtle et al. (2010a) investigated anthropometric variables in a small sample of female and male half-marathoners. They found significant differences in body mass, body height, body mass index, in skinfold thickness (*i.e.* triceps, thigh and calf) and in percent body fat.

Another important finding was that the sum of eight skinfold thicknesses was different between men and women since the sum of eight skinfold thicknesses was lower for men than for women. However, in keeping with Knechtle et al. (2010a), the sum of skinfolds was not different between men and women. We assume these differences are due to the larger sample size of subjects. Other more recent studies investigated 84 male half-marathoners (Rüst et al. 2011) and 42 female half-marathoners (Knechtle et al. 2011a). The present study, however, included twice the number of the subjects with 147 men and 83 women. In the current study and in the study of Knechtle et al. (2010a) investigating 52 men and 15 women, men had highly significantly thinner skinfolds (*i.e.* triceps, thigh and calf) than women. This might be due to the fact that men have a lower percent body fat. All other variables such as age, thickness of skinfolds at chest, midaxillary, abdomen and suprailiac site showed no significant sex differences.

Concerning training characteristics, men were running faster during training than women, had a faster personal best race time in half-marathon running and had completed more half-marathons than women. Besides, in the study of Knechtle et al. (2010a) investigating differences in anthropometric and training characteristics in female and male half-marathoners, men were running significantly faster during training sessions than women, but there were no significant differences in the number of previously finished half-marathons, and personal best times were not reported. Training characteristics were more predictive in women than in men for half-marathon race time. In the present subjects, running speed during training explained 34% of the variance in men and 59% in women. For example, for female marathoners, Hagan et al. (1987) developed an equation where marathon performance time (MPT) might be predicted (r = 0.82, r^2^ = 0.68) by the equation MPT (min) = 449.88 - 7.61 (km × day^-1^ run) - 0.63 (m × min^-1^, training pace). For male marathoners, physiological characteristics and age seemed also important (Hagan et al. 1981) since MPT may be predicted (r^2^ = 0.71) by the equation MPT (min) = 525.9 + 7.09 (km × workout^-1^) - 0.45 (training pace, m × min^-1^) - 0.17 (total km for 9 weeks) - 2.01 (VO_2_max, ml × kg^-1^ × min^-1^) -1.24 (age, years). Interestingly, also in these studies, the coefficient of determination of the models was higher in women (Hagan et al. 1987) than in men (Hagan et al. 1981).

We used percent body fat and running speed during training sessions since these variables were the best predictor variables for half-marathon race time in both men and women (Friedrich et al. 2014). Velocity during training seems to be a strong predictor variable for runners since running speed during training was also predictive for female (Schmid et al. 2012) and male (Barandun et al. 2012;Tanda and Knechtle 2013) marathoners. Regarding anthropometric characteristics, percent body fat was predictive for male marathoners (Barandun et al. 2012) and the circumference of the calf for female marathoners (Schmid et al. 2012). For male marathoners, race time for recreational might be partially predicted by the equation (r^2^ = 0.44) race time (min) = 326.3 + 2.394 × (percent body fat, %) – 12.06 × (running speed during training, km/h) (Barandun et al. 2012). The model including anthropometric and training variables explained 44% of the variance of marathon race times, whereas running speed during training sessions alone explained 40%. Therefore, also for male marathoners, running speed during training sessions was more predictive for marathon race times than anthropometric characteristics (Barandun et al. 2012). For female marathoners, race time might be partially (r^2^ = 0.50) predicted by the equation race time (min) = 184.4 + 5.0 × (circumference calf, cm) –11.9 × (speed in running during training, km/h) (Schmid et al. 2012). Again, also in these studies, the coefficient of determination of the models was higher in women (Schmid et al. 2012) than in men (Barandun et al. 2012). The present equations might be more feasible since for both women and men percent body fat was the same anthropometric predictor variable.

We also investigated apart from the anthropometric and training variables whether age and previous experience (*i.e.* number of completed half-marathons and personal best time in half-marathon) bi-variately correlated to race time. For both men and women, age and the personal best time in half-marathon correlated to race time, but not the number of completed half-marathons. Age and personal best time have been shown as important predictor variables in marathoners (March et al. 2011) and ultra-endurance athletes (Knechtle et al. [Bibr CR14][Bibr CR16][Bibr CR17][Bibr CR18]). For ultra-marathoners, age has been shown as an important predictor variable in male 100-km ultra-marathoners (Knechtle et al. 2010c) where race time might be predicted using the equation 1085.60 - 36.26 × (training speed, km/h) - 1.43 × (training volume, km/week) + 2.50 × (age, years). Personal best time in a shorter race (*i.e.* personal best marathon time) has been reported as important predictor variable in 24-hour ultra-marathoners where performance in a 24-hour ultra-marathon might be predicted using the equation 234.7 + 0.481 (longest training session before the 24-hour run, km) - 0.594 (personal best marathon time, min) (Knechtle et al. 2011b). Personal best time of the same race distance has been shown as important predictor variable in Triple Iron ultra-triathletes (Knechtle et al. 2011c) and ultra-endurance mountain bikers (2011d). In contrast to personal best half-marathon time, the number of completed half-marathons was not related to performance in both women and men. Regarding other races, the number of completed races might be predictive in ultra-endurance performance. In ultra-triathlon, the number of completed Triple Iron ultra-triathlons was related to race time in a Deca Iron ultra-triathlon (Herbst et al. 2011). In ultra-cycling, the number of completed mountain bike races such as the ‘Swiss Bike Masters’ was not related to race time (Knechtle et al. 2011d).

### Limitations

A limitation in this field study is that laboratory based results such as maximum oxygen uptake (VO_2_max) (Bragada et al. 2010;Tolfrey et al. 2009), lactate values (Grant et al. 1997;Roecker et al. 1998;Tolfrey et al. 2009) and treadmill velocity (Stratton et al. 2009) were not included. The inclusion of physiological variables such as VO_2_max might better explain the variance in running performance. A further limitation is that readings of skinfold measurement were taken after 4 s following Becque et al. (1986) and not after 2 s (Kramer and Ulmer 1981) following ISAK (International Society for the Advancement of Kinanthropometry) conventions (http://www.isakonline.com/). This might have influenced the sum of skinfolds and body fat calculations.

## Conclusions

For recreational female and male half-marathoners, the present equations using percent body fat and running speed during training units might be simpler and better to predict half-marathon race time for women and men, however, the coefficients of determination of the models increased not by using larger samples and percent body fat for both women and men. Future studies might include physiological variables to increase the coefficients of determination of the models.
